# Treatment of Acute Flares of Chronic Pancreatitis Pain with Ultrasound Guided Transversus Abdominis Plane Block: A Novel Application of a Pain Management Technique in the Acute Care Setting

**DOI:** 10.1155/2014/759508

**Published:** 2014-09-25

**Authors:** Daryl I. Smith, Kim Hoang, Wendy Gelbard

**Affiliations:** ^1^Department of Anesthesiology, School of Medicine and Dentistry, University of Rochester, 601 Elmwood Avenue, P.O. Box 604, Rochester, NY 14642, USA; ^2^Department of Emergency Medicine, School of Medicine and Dentistry, University of Rochester, 601 Elmwood Avenue, P.O. Box 604, Rochester, NY 14642, USA

## Abstract

The use of transversus abdominis plane (TAP) block to provide either analgesia or anesthesia to the anterior abdominal wall is well described. The technique yields high analgesic effectiveness and is opioid sparing and potentially of long duration with reported analgesia lasting up to 36 hours. When compared to neuraxial analgesia, TAP blocks are associated with a lower incidence of hypotension and motor blockade. TAP blocks are typically described as providing somatic analgesia only without any effect on visceral pain. There may be, however, certain conditions in which TAP blocks can provide effective analgesia in pain of visceral or mixed somatic and visceral origin. We describe two cases in which TAP blockade provided complete control of pain considered to be of visceral origin.

## 1. Introduction

Acute flares of chronic pancreatitis have a reported incidence of 6-7 cases per 100,000 adults per year [1], occur approximately 3–5 times as frequently in males as in females [2], and contribute to the functional and morphologic loss of the gland [3]. Treatment of these episodes is costly with a reported average daily hospital expense of $1670.00 and a yearly estimated cost of $2.2 billion [4]. We have used transversus abdominis plane (TAP) blocks successfully to treat the pain of these episodes in two patients despite the previous consideration of the blocks to be effective only for somatic pain. The TAP blocks used in the acute care setting resulted in immediate persistent analgesia and allowed for rapid discharge from the emergency department.

## 2. Case Report 1

**Figure 1:**
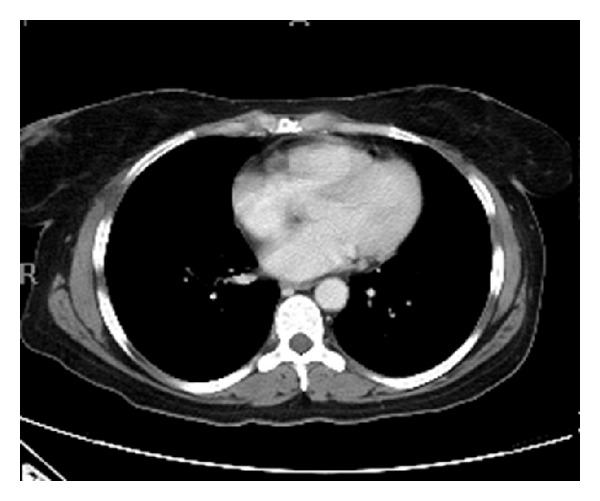
CT of abdomen in 46-year-old female with acute onset of abdominal pain with radiation of pain to back and history of pancreatitis.

The patient is a 46-year-old female with a 15-year history of chronic pancreatitis, hypertension, and alcohol abuse who reported to the emergency department with a four-day history of epigastric and right-sided abdominal pain consistent with her previous episodes of pancreatitis. The pain was constant in nature and aggravated by touch and movement; and physical examinationrevealed rebound tenderness. The pain radiated upwards to her sternum and traveled rightward and posteriorly to the muscles of her back. She complained of nausea, vomiting, and diarrhea and denied fever, chills, or headache. The patient stated that she had had a recent “flare” of pancreatitis treated with opiates in the emergency department 11 days earlier. She denied recent alcohol or recreational drug use but she had smoked one-third of a pack of cigarettes per day for 13 years. Computer assisted tomographic (CT) scan of the abdomen revealed an enlarged pancreas with adjacent fat stranding and fluid tracking along the retroperitoneum (Figure 1).

Intravenous narcotics were of limited benefit to the patient and she continued to report pain of 8/10 on the visual analog scale (VAS). The patient received a right-sided, ultrasound-guided TAP block via a mid-axillary approach bolused with 30 mL of 0.5% bupivacaine and 150 *μ*g epinephrine. The patient reported pain relief within ten minutes of the block and complete analgesia persisted through the time of her discharge home from the emergency department, two hours following the injection. Follow-up telephone conversations at 2 and 7 days after discharge revealed that the patient continued with complete pain relief (0-1/10 VAS) and she stated that she had not needed narcotics during that period.

## 3. Case Report 2

**Figure 2:**
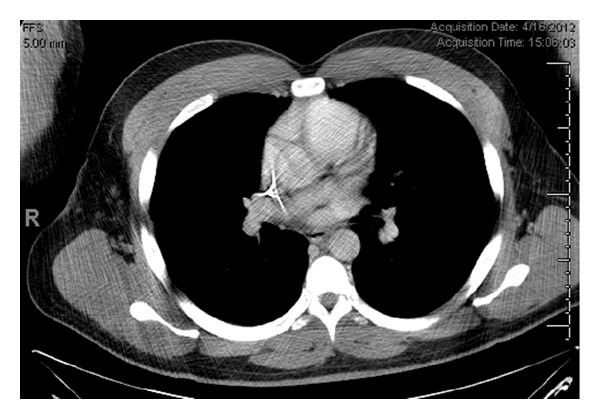
CT of abdomen in 30-year-old male with history of gallstone pancreatitis and pseudocyst with acute onset of abdominal pain.

The patient is a 30-year-old male with a history of gallstone pancreatitis and an infected pancreatic pseudocyst who presented with severe acute-on-chronic pancreatic pain. The pain was located in the left upper abdominal quadrant and periumbilical regions and was stated to be identical to past attacks of pancreatitic pain. In the 24 hours prior to admission the patient had taken 120 mg long-acting oxycodone and 80 mg immediate acting oxycodone with little effect. He denied nausea or vomiting but did have ongoing diarrhea. His physical exam was positive for abdominal tenderness but there was no rebound tenderness. The patient denied any history of ethanol use. He had undergone a laparoscopic cholecystectomy sixteen months prior to presentation. Abdominal CT revealed dilatation of the main pancreatic duct, pseudocyst of the pancreatic head, and coarse pancreatic parenchymal calcifications consistent with pancreatitis (Figure 2).

Intravenous narcotics were of limited benefit to the patient and he continued to report a pain level of 7-8/10 on the visual analog scale (VAS). An ultrasound-guided TAP block with 20 mL of a combination of 0.25% bupivacaine and 40 mg of depomedrol was performed. The patient reported pain relief within 5 minutes and was discharged home approximately one hour following the block. At 10-day telephone follow-up he reported that he had enjoyed complete relief of his symptoms for 7 days. He described a return of symptoms but his VAS pain score had reduced to 1-2/10.

## 4. Discussion

Transversus abdominis plane (TAP) blocks provide either analgesia or anesthesia to the anterior abdominal wall [5]. The technique yields high analgesic effectiveness, is opioid sparing [6], and is potentially of long duration with reported analgesia lasting up to 36 hours [7]. TAP blocks are typically described as providing somatic analgesia only without any effect on visceral pain yet there may be certain conditions in which TAP blocks provide effective analgesia in pain of visceral or mixed somatic and visceral origin.

We have used TAP blocks successfully to treat the pain of acute flares of chronic pancreatitis despite the previous consideration of the blocks to be effective only for somatic pain (Table 1). Initially we intended to alleviate the somatic component, the rebound and nonrebound abdominal wall tenderness [8], as there is at least one description of TAP block being used to provide analgesia for a patient with peritonitis [9], and the ability of the block to adequately anesthetize the abdominal wall [10]. The block, however, alleviated all components of pain in these patients. This included referred pain which radiated to the mid-back and vague aching that we considered to be of visceral origin. The patients were discharged home within 90–120 minutes of the injection and remained pain free at seven to ten days by telephone follow-up. We have also used TAP blocks on patients with viscerosomatic pain on an inpatient basis as well. These patients have experienced similar positive results (unpublished data). We focus here upon the emergency department experience because of the possible expediting of patient comfort and the potential cost benefit.

Pain is classified as visceral or somatic in origin to the exclusion of certain analgesic interventions in many instances. These distinctions may not be as clear cut as previously thought. Immunohistochemical and electrophysiologic studies have demonstrated significant overlap of somatic and visceral afferents in the dorsal horn of mammalian species. Spinal interneuron crosstalk and associated potentiation or depression at this level suggest that patterns of nociception may be mutually influential [11]. In addition serum levels of local anesthetics resulting from neural blockade have also been suggested to influence nociceptive pathway formation [12–14] and subsequent analgesic effect.

This case discussion addresses abdominal pain patterns usually considered to be of visceral origin and in this light it raises questions regarding the feasibility of categorically separating origins of pain with the goal of directing therapy. This is even more important as newer therapeutic interventions arise that may have a greater spectrum of action than previously thought. As ongoing* in vitro *studies elucidate the mechanisms involved in acute and chronic pain and the characteristics of neuronal plasticity, we may witness a significant expansion in therapeutic options available to patients suffering from both somatic and visceral pain syndromes. Both clinical and basic science researches are essential to the development of these treatment options.

TAP blockade is an easy to learn technique and its safety is enhanced by the use of ultrasound guidance. The blocks can also be placed with direct vision as in the intraoperative setting as well as with a “blind” or anatomic technique [15, 16] although recently there has been controversy regarding the accuracy of needle placement using this method [17]. In addition the occurrence of femoral nerve palsy resulting from spread of local anesthetic into the femoral nerve sheath during placement of the block using a “blind” technique has also been reported [18].

The cost saving of using TAP blocks in the acute care setting has yet to be determined. With our patients treated in the Emergency department as described in this report and with others treated on the hospital in-patient wards, discharge home quickly followed placement of the blocks and the onset of analgesia. The implications of this observation suggest that randomized control trials examining time to discharge following the use of TAP blocks in the acute care setting are warranted.

## Figures and Tables

**Table 1:** Summary of laboratory values and CT scan results in two patients treated with TAP blocks.

Case	Laboratory values (normal range)		CT scan of abdomen
	Lipase (28–100 U/L)	Amylase (13–60 U/L)	Results
1	48	65	(1) Enlarged pancreas (2) Fat stranding adjacent to pancreas (3) Fluid tracking along the retroperitoneum (4) Acute Pancreatitis

2	20	54	(1) Fluid density lesion in tail
			(2) Pancreatic edema in body
			(3) Pancreatic pseudocyst
